# High-Power Short-Duration Ablation of Paroxysmal and Persistent Atrial Fibrillation

**DOI:** 10.31083/j.rcm2412363

**Published:** 2023-12-25

**Authors:** Carolina Hoyos, Carlos D. Matos, Andres F. Miranda-Arboleda, Carlos Patino, Daniela Hincapie, Jose Osorio, Paul C. Zei, Jorge E. Romero

**Affiliations:** ^1^Cardiac Arrhythmia Service, Division of Cardiovascular Medicine, Brigham and Women’s Hospital, Harvard Medical School, Boston, MA 02115, USA; ^2^HCA Electrophysiology, Mercy Hospital, Miami, FL 33133, USA

**Keywords:** atrial fibrillation, pulmonary vein isolation, high-power short-duration, radiofrequency ablation, ablation techniques

## Abstract

Catheter ablation has become a cornerstone in atrial fibrillation (AF) therapy, improving freedom from all-atrial arrhythmias, as well as outperforming antiarrhythmic drugs in alleviating AF-related symptoms, reducing hospitalizations, and enhancing quality of life. Nevertheless, the success rate of traditional radiofrequency ablation (RFA) methods remains less than ideal. To address these issues, refinement in RFA strategies has been developed to improve efficacy and laboratory efficiency during pulmonary vein isolation (PVI). High-power short-duration (HPSD) RFA has emerged as a safe strategy to reduce the time required to produce durable lesions. This article reviews critical aspects of HPSD ablation in the management of both paroxysmal and persistent AF, covering aspects such as effectiveness, safety, procedural intricacies, and the underlying biophysics.

## 1. Introduction

Atrial fibrillation (AF) stands out as the most common sustained arrhythmia globally, affecting approximately 33.5 million people [1]. This prevalence has been consistently increasing, which could be attributed to the rise in life expectancy and multiple cardiovascular risk factors like hypertension, congestive heart failure, coronary artery disease, valvular heart disease, diabetes mellitus, obesity, and excessive alcohol use [1]. Catheter ablation (CA) is the cornerstone therapy in patients with symptomatic paroxysmal and persistent AF, significantly reducing all-atrial arrhythmias recurrence, AF-related symptoms, and hospitalizations, while improving quality of life [2, 3, 4].

Over the last decade, CA of AF has significantly enhanced safety and efficacy, owing to advancements in equipment and ablation techniques. The introduction of irrigated contact force (CF)-sensing catheters arose as an essential tool to improve the long-term safety and efficacy of CA of AF [5]. Moreover, a stable contact force, defined as >90% of the lesions created with a CF ≥10 g, was found to increase the probability of successful pulmonary vein isolation (PVI) at a 12-month follow-up [6]. Similarly, wide antral circumferential ablation (WACA) has proven to be more effective than ostial ablation with a lower rate of pulmonary vein stenosis [7, 8]. More recently, strategies such as high-frequency low-tidal volume ventilation, high-frequency ventilation, and high-frequency jet ventilation during CA of AF have improved long-term clinical outcomes and lesion durability [9, 10]. Despite these innovations, the success rate of AF ablation remains suboptimal, ranging from 74% to 86%. Patients undergoing repeated ablations often experience electrical reconnection of pulmonary veins (PVs) and the posterior wall (PW) at rates varying from 29% to 50% [11, 12, 13]. As such, there have been ongoing efforts to create transmural and durable lesions.

First described in 2006, high-power short-duration (HPSD) radiofrequency ablation (RFA) has shown advantages over traditional RFA settings in treating AF [14]. While conventional RFA typically uses 25–30 W for 30 seconds or more per lesion, HPSD RFA employs at least 40 W for less than 30 seconds per lesion [15]. The latter approach has proven effective in reducing both procedural and total radiofrequency (RF) times, while improving first-pass isolation rates, all without increasing complications [16].

## 2. Biophysics of HPSD radiofrequency ablation

RFA creates thermal lesions in cardiac tissue through alternating current, typically around 500 kHz, flowing from the catheter tip to a patch on the patient’s skin. The objective is to elevate tissue temperature to approximately 50 °C, inducing myocardial damage or necrosis. This process unfolds in two sequential stages: the resistive phase and the conductive heating phase. In the resistive phase, tissue directly in contact with the catheter heats up, affecting only a 1–2 mm radius from the catheter. This phase establishes a heat source that then progresses deeper into the tissue during the conductive phase, until the heat is dissipated. The lesion size is proportional to the temperature at the tissue contact point, with the highest temperature achieved during the resistive heating phase. As the heat conducts further into surrounding normothermic tissue both during and even after RF application, its effects dissipate over time. Therefore, by reducing the duration of RF application, the conductive heating impact on adjacent tissue is minimized [17]. This leads to more delimited lesion formation, potentially decreasing complications.

**Fig. 1.**
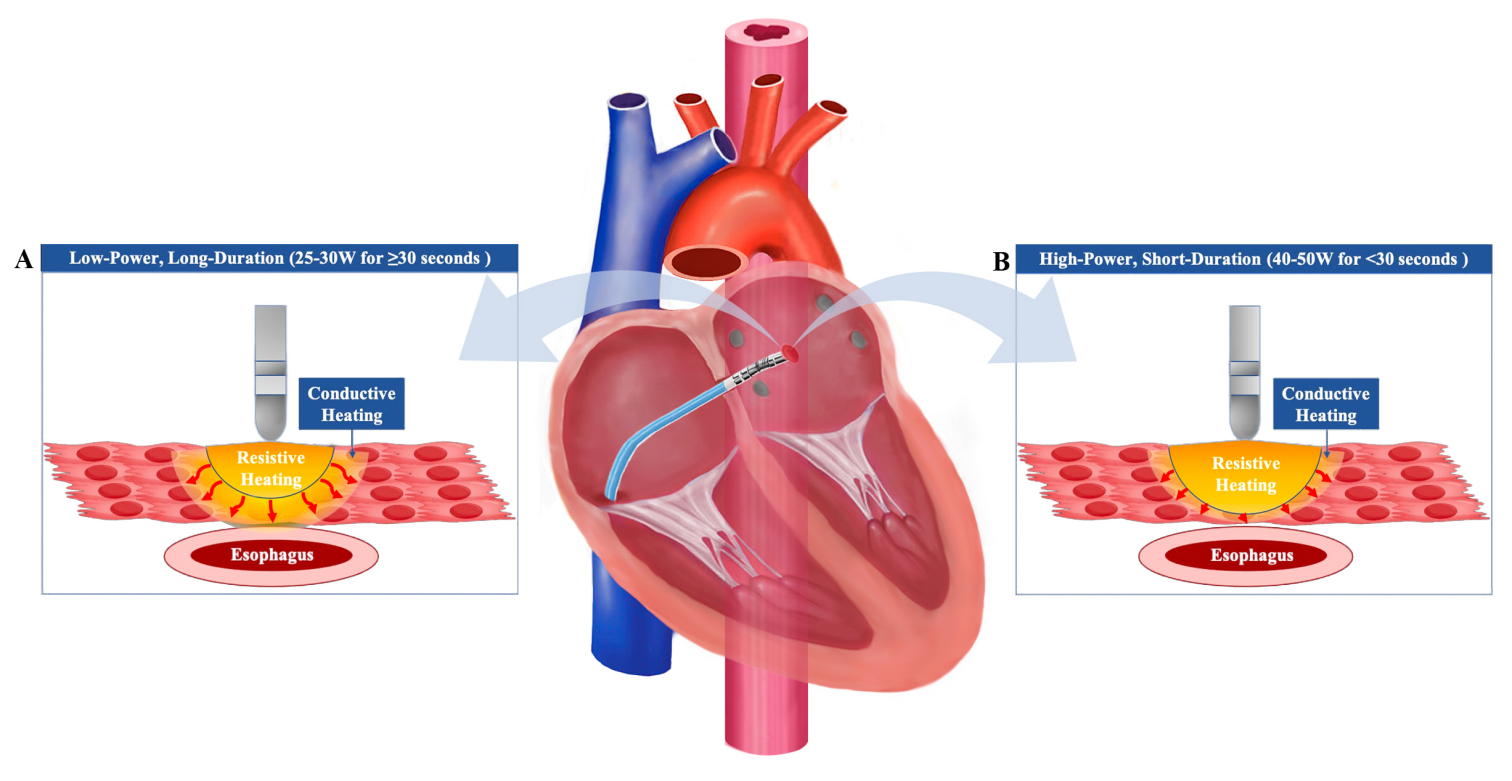
**Impact of Low-Power Long-Duration and High-Power Short-Duration Techniques on Tissue Heating**. Low-power long-duration (LPLD) ablation increases the conductive heating to adjacent tissues, such as the esophagus, while the resistive heating is limited (A). Conversely, high-power short-duration (HPSD) ablation increases the resistive heating while decreasing the conductive heating, potentially reducing the risk of thermal injury (B).

Traditional RFA involves 25–30 W of energy delivery for 30 seconds or more, usually described as low-power long-duration (LPLD) ablation. This prolonged heating extends conductive warmth to adjacent tissues, increasing the risk of thermal injury to nearby structures such as the esophagus and phrenic nerves. Conversely, HPSD RFA, which uses at least 40 W for less than 30 seconds, offers a different thermal profile. It produces a larger zone of higher temperature during the resistive phase but limits the duration of the conductive phase (Table 1, Fig. 1) [18]. The improved balance between resistive and conductive heating results in a more immediate and deeper lesion, while reducing passive heat spread to surrounding tissues. This aspect is particularly crucial as it reduces the risk of unintended heating of neighboring structures, thereby potentially mitigating the risk of esophageal, phrenic nerve, or even coronary artery injury. Leshem *et al*. [19] demonstrated that HPSD ablation was associated with significantly wider lesions (6.02±0.2 mm vs. 4.43±1.0 mm; *p* = 0.003) without affecting lesion depth (3.58 ± 0.3 mm vs. 3.53±0.6 mm; *p* = 0.81), when compared to LPLD ablation. This not only indicates the effectiveness of HPSD ablation but also implies enhanced uniformity among lesions.

**Table 1.** **Differences between HPSD vs. LPLD for the management of Paroxysmal and Persistent AF**.

High-Power Short-Duration Ablation	Low-Power Long-Duration Ablation
Energy delivery/Power levels: ≥40 watts.	Energy delivery/Power levels: 25–30 watts.
Ablation duration: <30 seconds per lesion.	Ablation duration: ≥30 seconds per lesion.
Lesion formation: thermal injury, wider transmural lesions.	Lesion formation: thermal injury, narrower lesions with major lesion depth.
Tissue cooling: use of specialized cooling techniques to prevent excessive tissue heating during higher-energy bursts. Less risk of thermal injury to adjacent structures.	Tissue cooling: achieved through the use of irrigation systems. Higher risk of thermal injury to adjacent structures.
Learning curve: requires specific training due to its status as a relatively novel and less commonly disseminated technique.	Learning curve: its use is more established and widely adopted by electrophysiologists around the world.

Abbreviations: HPSD, high-power short-duration; LPLD, low-power long-duration; AF, atrial fibrillation.

## 3. Radiofrequency Settings for HPSD Ablation

The choice of RFA settings serves as an essential step in optimizing safety and efficacy outcomes. Since the introduction of generators with power-controlled mode and irrigated catheters, maintaining constant power has become more manageable. However, lesion-to-lesion dimensions can still vary significantly due to dynamic current adjustment based on baseline impedance. A study by Barkagan *et al*. [20] demonstrated an inverse relationship between baseline impedance and the square of the current. Keeping a low baseline impedance allows for a higher current flow into the myocardium, which consequently produces larger lesions [20]. Preliminary studies have determined that doubling the surface area of the return skin patch, while decreasing the distance between this skin patch and the catheter tip, reduces the baseline impedance. This is attributed to the high variability of the baseline impedance based on the amount of fat or air and the location of the dispersive electrode [20, 21]. To mitigate this variability, it is recommended to place two patches in a zone with a minimal volume of subcutaneous fat while properly cleaning the skin where the return skin patches will be applied, avoiding air bubbles.

Maintaining an optimal temperature at the catheter-tissue interface is critical to prevent complications such as char formation or steam-pops. The target temperature should not exceed 50 °C. To achieve this, it is essential to adjust the irrigation flow rate and accurately measure the tissue temperature, taking into consideration the cooling effects of the irrigation fluid during the ablation process. In the context of HPSD RFA, the SmartTouch Surround flow® (STSF; Biosense Webster, Inc. Irvine, CA, USA) allows a safe delivery of up to 50 W of power with an irrigation flow rate of 15 mL/min [22]. When using Tacticath SE® (Abbott, Chicago, IL, USA) or the Intellanav Stablepoint (Boston Scientific, Marlborough, MA, USA), the irrigation flow rate should be increased to 30 mL/min [23]. These specific flow rates and catheter choices serve to optimize the balance between safety and efficacy in HPSD RFA procedures.

A study by Ali-Ahmed *et al*. [24] demonstrated that 50 W-HPSD ablation achieves a suitable temperature at a 5-mm depth in the myocardium, potentially reducing the risk of thermal injury of neighboring structures. This article also showed that achieving a 4-mm lesion required 20 seconds using a power of 20 W compared to just 6–7 seconds using a power of 50 W [24]. This indicates that similar lesion sizes can be accomplished with both power settings, but with a shorter energy delivery time in the 50 W setting [24]. Accordingly, establishing a maximal ablation time and adequate temperature monitoring may help to reduce the risk of myocardial perforation and collateral damage.

**Fig. 2.**
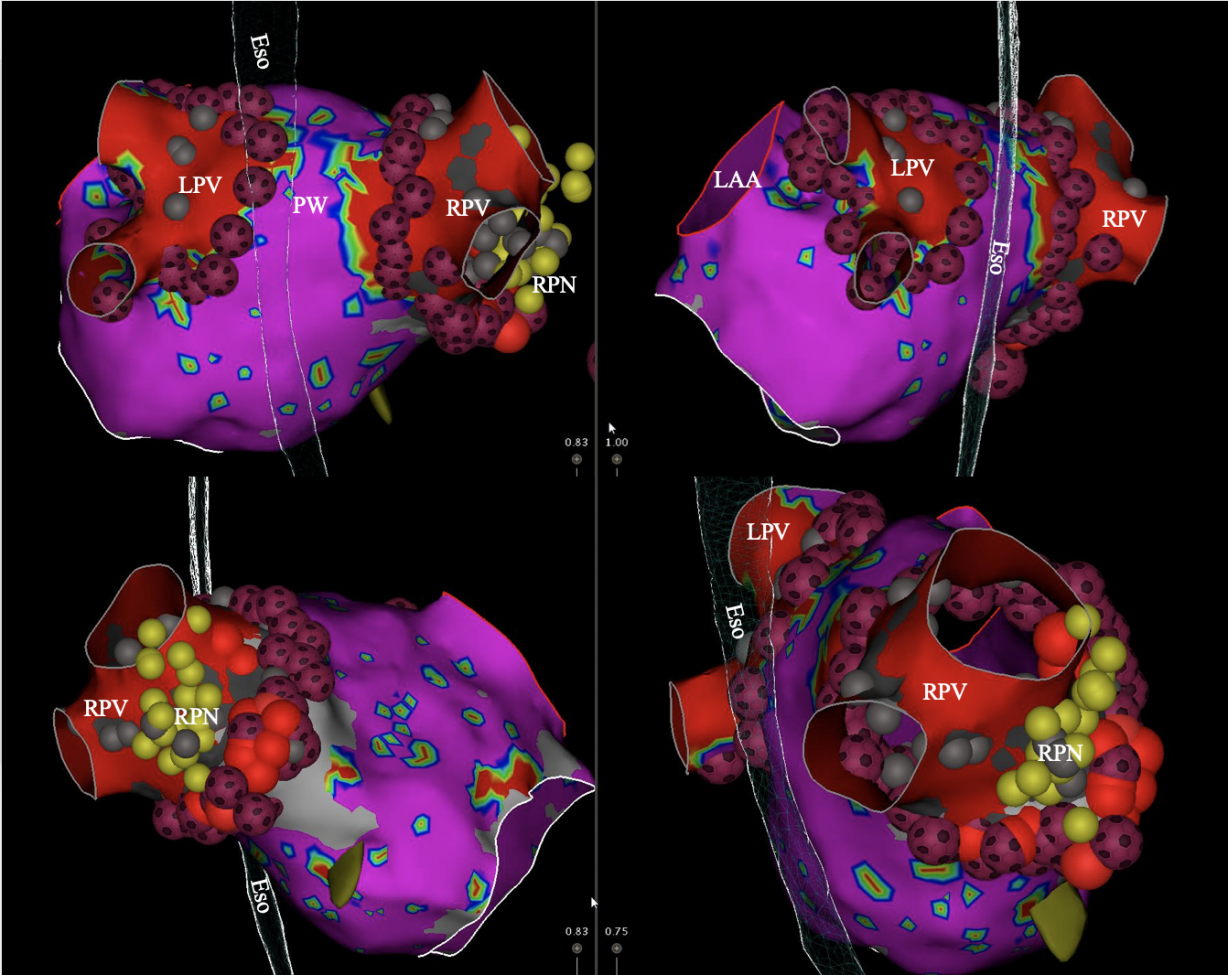
**Voltage map of the left atrium showing pulmonary vein isolation (PVI) with QDOT ${\mathrm{MICRO}}^{\text{𝐓𝐌}}$ catheter (Biosense Webster, Inc. Irvine, CA, USA)**. Abbreviations: LPV, left pulmonary veins; Eso, esophagus; RPV, right pulmonary veins; RPN, right phrenic nerve; LAA, left atrial appendage; PW, posterior wall. *Used with permission from Cardiotext Publishing. Amin Al-Ahmad. Hands-On Ablation: The Expert’s Approach. Third Edition. Minneapolis, MN: Cardiotext; December 2022.*

The novel QDOT ${\mathrm{MICRO}}^{\text{TM}}$ catheter (Biosense Webster, Inc. Irvine, CA, USA) was developed to deliver very high-power short-duration (vHPSD; i.e., 90 W for 4 seconds) and temperature-controlled ablation. This catheter incorporates six thermocouples in the outer metal shell. These thermocouples allow for dynamic control of both irrigation flow and power based on real-time temperature data, ensuring a constant tissue-catheter interface temperature of around 65–70 °C during ablation (Table 2, Ref. [19, 25]; Fig. 2). When using this strategy, the RF generator should be set correspondingly, and the CF should be kept between 5 g to 30 g. Additional touch-ups should be performed using 50 W [25]. A recent multicenter study demonstrated that 86% of cases achieved clinical success with vHPSD ablation, while 92.1% avoided the need for repeat procedures [26]. Although there is no standardized power setting to perform HPSD ablation, most studies have reported the use of 40 W, 45 W, 50 W, and 90 W [16, 25, 27, 28]. Despite the promise of 90 W settings, its use is constrained by the availability of specialized equipment and no marked long-term outcomes difference has been shown when compared to 50 W. Therefore, HPSD ablation using power levels between 40–50 W is currently the more practical choice.

**Table 2.** **Comparison of the catheters that can be used for HPSD Ablation for Paroxysmal and Persistent AF**.

Type of Catheter	Advantages	Disadvantages
Temperature-controlled catheters	Reduces procedural time by almost 90 minutes and lowers procedure cost without compromising safety.	Their use requires specialized training and expertise.
-QDOT ${\mathrm{MICRO}}^{\text{TM}}$ Catheter	Improved Proximal Irrigation.	Longer learning curve.
(Biosense Webster Inc. Irvine,	Improved Temperature Monitoring.	Higher costs.
CA, USA)	Higher Signal Resolution.	Limited availability.
	Controlled energy delivery with lower rates of complications such as steam pops and perforations during ablation.	Lack of long-term data about the durability of ablation lesions.
	Delivers very high-power short-duration (vHPSD; i.e., 90 W for 4 seconds) and temperature-controlled ablation.	
	Constant temperature (i.e., 65–70 °C) [19].	
	Seamlessly integrated with the CARTO® 3 System, which combines contact force technology, 3D mapping, and advanced navigation capabilities [25].	
Irrigated contact force conventional RF catheters	Provides consistent movement in response to contact force using precision spring, promoting consistent lesion formation.	Risk of fluid accumulation in the heart chambers with the irrigation.
-THERMOCOOL SMARTTOUCH® SF Catheter - STSF	Sends accurate location reference signal via location sensor and transmitter coil.	More complexity set up with the irrigation system.
(Biosense Webster Inc. Irvine, CA, USA)	Reduces ablation time.	Higher risk of thrombus formation and secondary embolic events.
-${\mathrm{TACTIFLEX}}^{\text{TM}}$ Catheter (Abbott, Chicago, IL, USA)	Improves outcomes with more stable catheter-tissue contact force and catheter tip direction.	Higher costs due to the additional components required for the irrigation system.
	Superior tip stability and excellent signal quality during ablation.	Limited access.
	Predictability with intuitive contact force arrow and deflection direction indicator available with the EnSite X EP System.	

Abbreviations: AF, atrial fibrillation; HPSD, high-power short-duration; vHPSD, very high-power short-duration; RF, radiofrequency; 3D, three dimensional.

## 4. Efficacy and Safety of HPSD Ablation

PVI durability is highly related to consistent transmural lesion formation [19]. Maintaining a stable CF during RF application significantly contributes to increasing lesion size and achieving lesion transmurality [29]. Consequently, HPSD ablation may reduce the time required to generate an effective lesion, decreasing the time during which catheter stability must be kept constant, and improving electrophysiology laboratory efficiency. Moreover, since HPSD ablation reduces the conductive heating, it leads to wider and shallower lesions than LPLD, making it preferable for thin tissue such as the atria [19]. Thus, HPSD ablation has been associated with a reduction in maximal esophageal temperatures during ablation of the posterior wall [30] and a reduced risk of phrenic nerve injury [31].

In a systematic review and metanalysis comparing LPLD ablation vs. HPSD ablation for either paroxysmal or persistent AF, HPSD ablation yielded significantly better results [32]. Specifically, it achieved a higher rate of freedom from all-atrial arrhythmias (odds ratio [OR]: 1.48, 95% confidence interval [CI]: 1.12–1.94, *p* = 0.005) and was more effective in first-pass PVI (OR: 8.92, 95% CI: 2.40–33.09, *p* = 0.001) [32]. Importantly, there was no significant increase in procedural complications [32]. Likewise, in a randomized clinical trial including 60 patients undergoing catheter ablation of paroxysmal or persistent AF, HPSD ablation resulted in a significant reduction in atrial arrhythmias recurrence (10% vs. 35%; hazard ratio [HR]: 0.26; *p* = 0.027) and shorter time to achieve PVI (87 minutes vs. 126 minutes; *p = *0.003) compared to LPLD ablation [33]. However, large-scale randomized trials are needed to unequivocally establish the improved efficacy and safety outcomes linked to HPSD ablation.

**Fig. 3.**
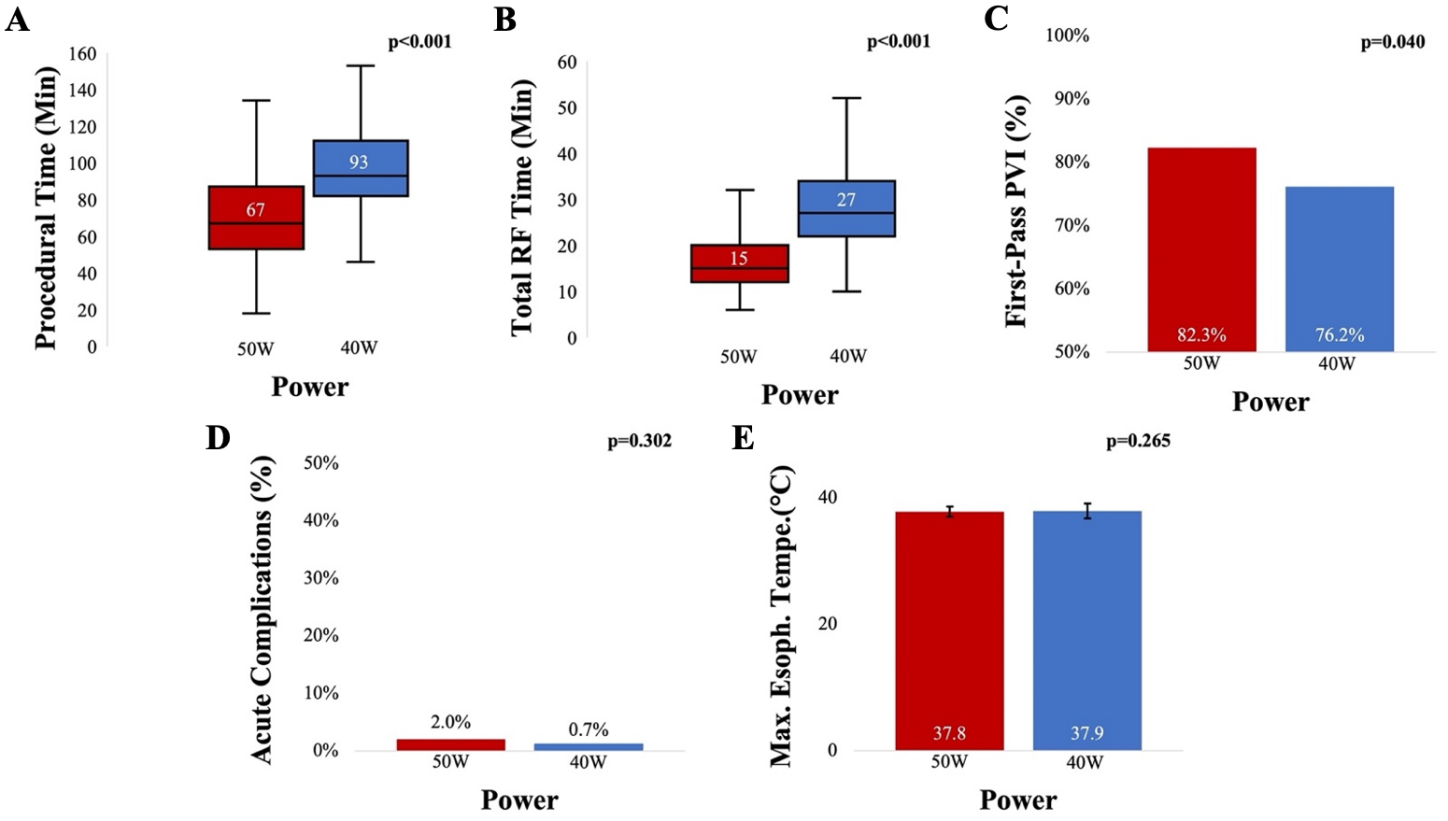
**Comparing Ablation Strategies in Atrial Fibrillation: 50 W-HPSD vs. 40 W-HPSD - Procedural Efficiency and Outcomes**. 50 W-HPSD ablation was associated with a significantly shorter ablation and procedural time compared to 40 W-HPSD ablation (A, B). The rate of first-pass isolation was significantly higher in the 50 W-HPSD ablation group than in the 40 W-HPSD ablation group (C). No difference was observed in maximum esophageal temperature and acute complications between groups (D, E). Abbreviations: HPSD, high-power short-duration; Min, minutes; PVI, pulmonary vein isolation; RF, radiofrequency; Max. Esoph. Tempe, maximal esophageal temperature. *Used with permission from Elsevier. Costea et al. 50 W vs. 40 W During High-Power Short-Duration Ablation for Paroxysmal Atrial Fibrillation: A Multicenter Prospective Study. Journal of the American College of Cardiology: Clinical Electrophysiology. in PRESS.*

In a recently published multicenter study comparing the safety and efficacy of power settings in HPSD ablation, findings suggest comparable outcomes for both 40 W-HPSD and 50 W-HPSD ablation in patients treated for paroxysmal AF [34]. Both power settings demonstrated similar 12-month freedom from all-atrial arrhythmias, procedural complications, and maximum esophageal temperatures. However, 50 W-HPSD ablation was associated with shorter procedural and RF times as well as higher rate of first-pass PVI (Fig. 3) [34]. Moreover, a systematic review and metanalysis, including studies that compared vHPSD vs. HPSD ablation, showed similar efficacy and safety outcomes between the two approaches [35]. In preclinical studies, tissue temperatures achieved with HPSD ablation guided by ablation index (AI) have been reported as similar to those obtained with vHPSD ablation [36]. In summary, while all forms of HPSD ablation appear to be relatively safe and effective, subtle advantages may exist depending on the specific power setting used.

During CA for AF, thrombus formation remains a significant concern despite the routine use of intraprocedural heparinization, determined by increased activated clotting time (ACT) levels of at least 300–350 seconds [37]. Notably, the incidence of asymptomatic cerebral emboli (ACE) has been reported to be around 13%, but may vary according to the imaging technique and definition [37]. A silent cerebral event (SCE) is defined as positive diffusion-weighted imaging (DWI) without fluid-attenuated inversion recovery (FLAIR) and is not associated with cell death. In contrast, a silent cerebral lesion (SCL) is described as positive DWI with FLAIR due to the edema caused by cell death. While SCEs may be detected within 24–72 hours after the procedure, SCLs may be found up to seven days after the ablation [38]. A recently published randomized controlled trial comparing HPSD vs. LPLD ablation demonstrated a non-significant trend of higher ACE in patients undergoing HPSD RFA compared to those in the LPLD group (40% vs. 17%; *p* = 0.053) [33]. This trend aligns with previous preclinical studies associating high-power standard-duration ablations (i.e., 50 W for 30 seconds) to a higher risk of microembolic events compared to standard RF ablation power settings [39]. In a substudy of the AXAFA-AFNET 5 Trial (Anticoagulation Using the Direct Factor Xa Inhibitor Apixaban During Atrial Fibrillation Catheter Ablation: Comparison to Vitamin K Antagonist Therapy), which included 335 patients undergoing brain magnetic resonance imaging (MRI) after AF ablation, acute brain lesions were detected in 25% of the patients [40]. Notably, there were no differences in cognitive function between patients with and without acute brain lesions [40]. Further clinical studies are needed to assess the association of ACE with HPSD ablation, as well as the the short- and long-term endpoints.

## 5. Ablation Endpoints

**Fig. 4.**
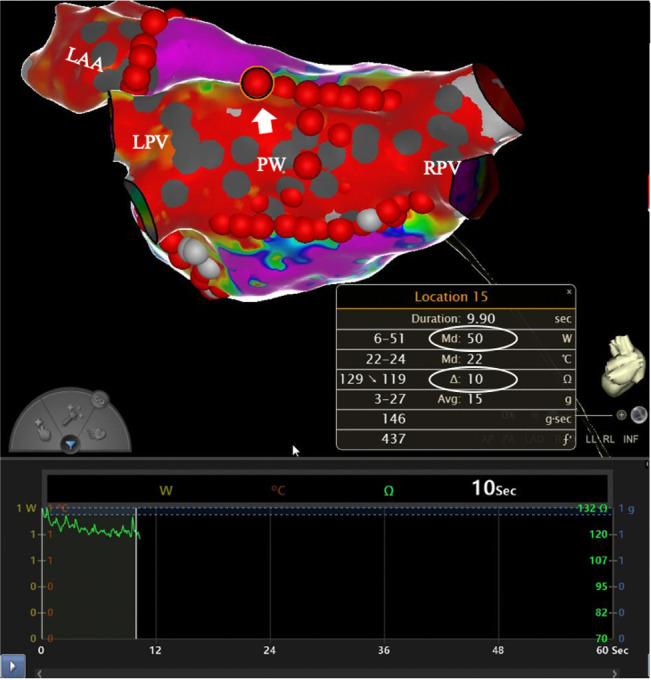
**Target impedance drop of 10 ohms and contact force of 15 g achieved with 50 W-HPSD ablation**. Abbreviations: LAA, left atrial appendage; LPV, left pulmonary veins; PW, posterior wall; RPV, right pulmonary veins; HPSD, high-power short-duration. *Used with permission from Cardiotext Publishing. Amin Al-Ahmad. Hands-On Ablation: The Expert’s Approach. Third Edition. Minneapolis, MN: Cardiotext; December 2022.*

A well-established protocol should be followed to obtain improved efficacy and safety outcomes. Setting appropriate parameters on the RF generator is essential to avoid excess exposure, limiting the maximum RF application time to a few seconds, which is in line with the HPSD ablation protocol. This can mitigate the risk of inadvertent prolonged high-power RF application, thereby lowering the likelihood of adverse events such as steam pops and myocardial perforation [24]. Additionally, maintaining a consistent CF between 10–20 g prior to initiating RF delivery is essential. Avoiding CF above 20 g is crucial to reduce the risk of esophageal injury in patients undergoing RF ablation. Moreover, to reduce the risks associated with AF ablation, the use of an esophageal temperature probe can serve as a continuous monitor, guiding RF delivery. Lesion overlap should be avoided to reduce the risk of conductive heating of adjacent tissues. However, lesion contiguity is vital; a distance of less than 6 mm between individual lesions should be preserved. Targeting an impedance drop of 10–15 ohms per lesion is another crucial measure, which has been demonstrated to be a reliable indicator of ablation efficacy (Fig. 4) [41, 42]. During ablation procedures, standard lesion quality indexes should be used as guidance. When utilizing the CARTO 3 AI, specific AI values are recommended depending on the lesion’s location. For lesions in the anterior portion of the PVs, an AI of 550 is recommended, while an AI of 400 is suggested for lesions in the posterior portion [43]. Alternatively, if the EnSite lesion size index (LSI) is used, specific target values are recommended based on the lesion’s anatomical location. For lesions in the anterior segment of the PVs, a LSI of 5.2 is advised, while a target LSI of 4 is suggested for lesions in the posterior region [44]. These indexes help ensure appropriate lesion formation during the ablation process. In addition to these standardized indexes, the absence of the negative component in the unipolar atrial signal can also indicate effective ablation. This absence is thought to signify the creation of a transmural lesion, further supporting the procedure’s efficacy [45].

## 6. Adjunctive Ablation Strategies

Electrical isolation of PVs is recommended during all AF ablation procedures since the ectopic beats that induce AF are usually located in the PVs [46]. PVI is considered the pillar of the treatment of paroxysmal and persistent AF. Although the dissociation of PV potentials is the key endpoint, adjunctive ablation strategies have been embraced due to the high recurrence of atrial arrhythmia in patients with persistent AF. These additional ablation strategies include linear ablations in the left atrium (LA) or right atrium (RA), posterior wall isolation (PWI), LA appendage electrical isolation (LAAEI), ablation of non-PV foci, ablation of complex fractionated atrial electrograms (CFAE) and rotational activity.

### 6.1 Posterior Wall Isolation

Although PVI is the preferred ablation technique for patients with paroxysmal AF, its efficacy in patients with persistent AF is suboptimal. A significant proportion of patients (up to 57%) experience arrhythmia recurrence during follow-up [47]. A meta-analysis has reported the benefits of combining PVI with PWI in patients with persistent AF, showing a 26% relative risk reduction and 8% absolute risk reduction in atrial arrhythmia recurrence [48]. Nevertheless, recent randomized studies performed with RF have not confirmed these improvements in the freedom from atrial arrhythmia recurrence [49, 50]. Possible explanations for these controversial findings include the selection of healthier patients (i.e., patients with a lower burden of AF, smaller LA volumes, and shorter AF duration), in whom PVI only may be sufficient (even though they have been labeled as persistent based on current AF classification); as well as the creation of incomplete transmural lesions, leading to PW reconnection in 40% of patients and facilitating the creation of re-entrant circuits. Notably, there are technical challenges in performing a successful PWI, and the immediate proximity of the PW to the esophagus may preclude operators from performing adequate ablation lesions because prolonged RF delivery may increase the risk of an atrio-esophageal fistula formation [51]. However, multiple studies have shown similar safety outcomes in patients undergoing PWI with HPSD ablation vs. LPLD ablation, with a low occurrence of esophageal injury [30, 52]. To reduce the risk of esophageal thermal injury, a lower CF (<10 g) and a shorter RF delivery (i.e., 50 W for 5 seconds) are recommended during PWI using HPSD ablation.

### 6.2 Left Atrial Appendage Electrical Isolation

In 2005, Takahashi *et al*. [53] reported that a patient with paroxysmal AF had multiple foci identified in the left atrial appendage (LAA) after PVI. The patient was successfully treated with CA by disconnecting this structure electrically from the LA, underscoring the LAA’s role in arrhythmogenesis [53]. Supporting this, the BELIEF study (Effect of Empirical Left Atrial Appendage Isolation on Long-term Procedure Outcome in Patients With Persistent or Longstanding Persistent Atrial Fibrillation Undergoing Catheter Ablation) focused on patients with non-paroxysmal AF undergoing CA, and found that empirical LAAEI improved long-term procedure outcomes [54]. During a 12-month follow-up, a meta-analysis reported freedom from all-atrial arrhythmia recurrence of 75.5% in patients who underwent LAAEI vs. 43.9% in whom only standard ablation was performed (56% relative risk reduction and 31.6% absolute risk reduction; relative risk [RR] 0.44, 95% CI 0.31–0.64, *p* < 0.0001) without increasing acute procedural complications or embolic stroke risk [55].

**Fig. 5.**
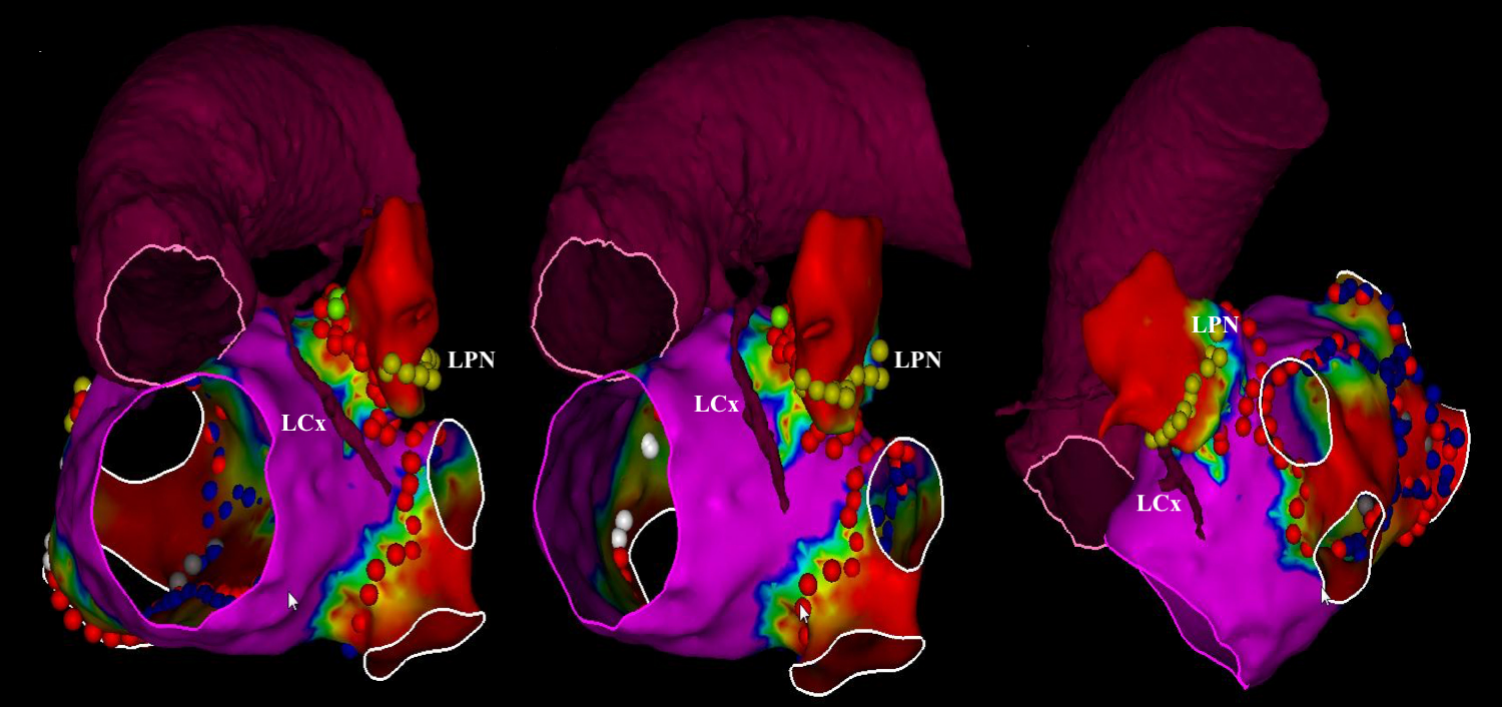
**Voltage map showing left atrial appendage electrical isolation**. Abbreviations: LCx, left circumflex; LPN, left phrenic nerve. *Reprinted from JACC: EP. 2020; 6: 157–167. Romero *et al*. Imaging Integration to Localize and Protect the Left Coronary Artery in Patients Undergoing LAAEI, with permission from Elsevier.*

LAAEI is performed by delivering RF energy at the level of the LAA ostium (Fig. 5). RF settings during LAAEI typically include a power of 45 W while maintaining a temperature of 42 °C for a maximum of 15–18 seconds per lesion. Nonetheless, longer lesions may be required for the anterior and superior edges of the LAA (which are known to be thicker than the inferior and posterior margins) [56]. The CF should be maintained between 15–30 g during LAAEI.

### 6.3 HPSD Ablation to Treat Mitral-Dependent Macro-Reentrant Arrhythmias

Evidence supports the advantages, safety, and efficacy of using HPSD ablation for creating circular lesions around the PV antra during PVI procedures [57, 58, 59, 60]. A growing body of research is focused on broadening the scope of applications of HPSD ablation to treat other types of arrhythmias and target additional areas within the LA. In particular, Zanchi *et al*. [61] explored the feasibility of performing anterior mitral lines (AML) and roof lines (RL) using a hybrid approach delivering HPSD lesions but aiming for a target AI of 500 for AMLs and 400 for RLs. A total of 35 patients were included, and 32 AMLs were performed. First-pass block was obtained in 75% of cases while gaps were mapped in 25% of patients, the latter showing the most common area for persistent conduction was the middle third of the line [61]. Acute success was reached in 97% (31/32) of patients with a RF time of 2.9 ± 0.8 minutes, a mean fluoroscopy time of 0.4 ± 0.6 minutes, and a mean AML length of 62 ± 9 mm [61]. Crucially, the study revealed no increase in complications, and although there was a 6% incidence of steam pops, they did not have clinical significance [61].

Case reports have described the QDOT Micro catheter (Biosense Webster, Irvine, CA, USA), with the ability to deliver temperature-controlled ablation with CF sensing. This catheter offers flexibility in delivering either conventional HPSD lesions at 50 W or vHPSD lesions at 90 W for 4 seconds [62]. In one notable case, an ablation procedure using the vHPSD configuration successfully terminated a mitral-dependent atrial flutter [63]. An AML was established with a bidirectional block in less than two minutes of RF activity [63]. During this case, 90 W for 4 seconds successfully created a cavotricuspid isthmus (CTI) line with a bidirectional block [63].

The DiamondTempTM (DT)-catheter, a new product from Medtronic designed for HPSD ablation in a temperature-controlled mode, was recently evaluated to create mitral isthmus lines (MIL) [64, 65]. This device successfully achieved bidirectional block during MIL in 19 out of 20 patients (95%) [64, 65]. The duration of applications varied between the groups, with times of either 10 seconds (group A) or 20 seconds (group B) [64, 65]. To accomplish a bidirectional block, 80% of patients in Group A and 50% of patients in Group B required endocardial lesions from the CS (coronary sinus) [65].

### 6.4 HPSD to Isolate the Superior Vena Cava

The superior vena cava (SVC) is one of the most common non-pulmonary vein triggers in patients with AF [66]. Although controversial, isolation of the SVC may be considered in some patients after PVI as it has been associated with potentially better procedure outcomes [67, 68, 69]. Nonetheless, the best methodology for SVC ablation has not yet been established. Considering the success of HPSD ablation in the LA and RA areas [57, 58, 59, 60], researchers have begun exploring the applicability of this technique to the SVC.

In a 2021 study by Kusa *et al*. [70], 100 patients undergoing PVI also received SVC isolation. The cohorts were evenly distributed, with 50 patients assigned to the HPSD ablation group (50 W for 4–7 seconds), and another 50 patients in the LPLD ablation group (20–25 W for 20–30 seconds) [70]. Acute SVC isolation was successfully achieved in 100% of patients in both groups. Nevertheless, the HPSD ablation strategy was associated with shorter RF times, fewer ablation lesions, and lower AIs [69]. Although numerically higher rates of phrenic nerve injury were observed in the LPLD ablation group (3 cases) compared to the HPSD ablation group (1 case), the difference was not statistically significant [70].

Due to the biophysical properties, HPSD lesions tend to be shallower with a wider diameter [71]. This characteristic makes HPSD ablation particularly advantageous for SVC ablations, as it minimizes collateral damage—especially near sensitive structures such as the phrenic nerves, without increasing short or long-term complications [31]. It is essential to account for the unique tissue characteristics at the SVC–RA junction; these regions are generally thinner than other areas in the LA [72]. Accordingly, the AI tends to be lower, with a mean of 379 in non-lateral segments and 345 in lateral segments [72]. Being aware of these specific values can reduce the risk of delivering unnecessary long lesions and will decrease the development of complications such as steam pops.

## 7. Conclusions

High-power short-duration RFA for AF relies mainly on resistive heating while limiting conductive heating; therefore, leading to a decreased risk of thermal injury to adjacent structures. To achieve optimal results, appropriate RF settings are essential, such as maintaining a constant CF, avoiding excessive baseline impedance, and adjusting irrigation flow rates. This technique allows the creation of shallower, wider, and more durable lesions, enhancing the success rate of PVI and reducing the risk of electrical reconnection.

Although HPSD ablation presents a compelling alternative to conventional LPLD ablation, offering advantages including improved efficacy, shorter procedural time, similar complication rates, and better lesion contiguity, uncertainties remain. The occurrence of ACE is one such concern, and further studies are needed to evaluate its long-term outcomes. Additionally, information from prospective registries and larger randomized trials are required to confirm the efficacy and safety of HPSD ablation compared to conventional methods.

## Data Availability

Not applicable.

## Abbreviations

ACE, asymptomatic cerebral emboli; ACT, activating clotting time; AF, atrial fibrillation; AI, ablation index; AML, anterior mitral lines; CA, catheter ablation; CF, contact force; CFAE, complex fractionated atrial electrograms; CTI, cavotricuspid isthmus; DWI, diffusion-weighted imaging; FLAIR, fluid-attenuated inversion recovery; HPSD, High-power Short-duration; LA, left atrium; LAA, left atrial appendage; LAAEI, left atrium appendage electrical isolation; LPLD, Low-power Longer-duration; LSI, lesion size index; MIL, mitral isthmus lines; PV, pulmonary veins; PVI, pulmonary vein isolation; PW, posterior wall; PWI, posterior wall isolation; RA, right atrium; RF, radiofrequency; RFA, radiofrequency ablation; RL, roof lines; SCE, silent cerebral event; SCL, silent cerebral lesion; SVC, superior vena cava; WACA, wide antral circumferential ablation.
